# Adulticide treatment with melarsomine: outcome in 283 heartworm-positive dogs in Germany

**DOI:** 10.1186/s13071-026-07404-2

**Published:** 2026-08-20

**Authors:** Imke Maerz, Sophie Rütjes, Sebastian Genz, Pia Žagar

**Affiliations:** 1 Tierklinik Stuttgart-Plieningen, AniCura Stuttgart GmbH, Stuttgart, Germany; 2 Tierklinik Hofheim, IVC Evidensia GmbH, Hofheim, Germany

**Keywords:** *Dirofilaria immitis*, Complications, Heat treatment, Echocardiography, Worm burden, Distensibility index

## Abstract

**Background:**

Heartworm disease is of increasing significance in Europe because of climate change and the movement of dogs from endemic regions. Despite guidelines from the American Heartworm Society (AHS) and the European Society of Dirofilariosis and Angiostrongylosis (ESDA), some clinicians favor macrocyclic lactones and doxycycline alone, partly because of concerns about melarsomine complications. Recent studies, however, support the safety and efficacy of the recommended protocol.

**Methods:**

This retrospective study analyzed the outcomes of dogs with Class I–III heartworm disease that underwent melarsomine adulticide treatment at a German referral center. Demographic, clinical, diagnostic, therapeutic, and outcome data, including adverse events, were systematically retrieved. Diagnosis was confirmed by antigen testing, with heat treatment applied when indicated. Therapy consisted of 28 days of doxycycline and monthly macrocyclic lactones, followed by a three-dose melarsomine protocol as recommended by AHS/ESDA. Supportive care included sedation, intravenous fluids, maropitant, and additional medications as clinically indicated.

**Results:**

A total of 283 dogs were included. Median age was 4.2 years (range: 8 months–11.5 years), and median body weight was 19 (range: 3.4–51.1) kg. Females comprised 53% (84% spayed) and males 45% (89% neutered). All dogs originated from endemic European countries. Clinical signs before treatment included respiratory symptoms (31%), exercise intolerance (15%), gastrointestinal signs (8.5%), and others (2%), while 53% were asymptomatic. Complications following melarsomine were generally mild: injection-site soreness (9.2% after first, 2.1% after third injection), gastrointestinal signs (3.5% after first, 0.7% after third injection), and coughing (12.7% after first, 5.3% after third injection). Two dogs (0.7%) died during treatment, one from cardiac arrest under anesthesia and one from pancytopenia, but neither was conclusively attributable to melarsomine. At 6-month follow-up, none of the 221 dogs tested remained antigen positive.

**Conclusions:**

Adulticide treatment with melarsomine showed high efficacy and an acceptable safety profile. Most adverse events were mild and self-limiting, with rare severe complications. These findings support the use of evidence-based, guideline-driven protocols to manage heartworm disease in dogs living in European countries.

**Graphical Abstract:**

**Figure:**
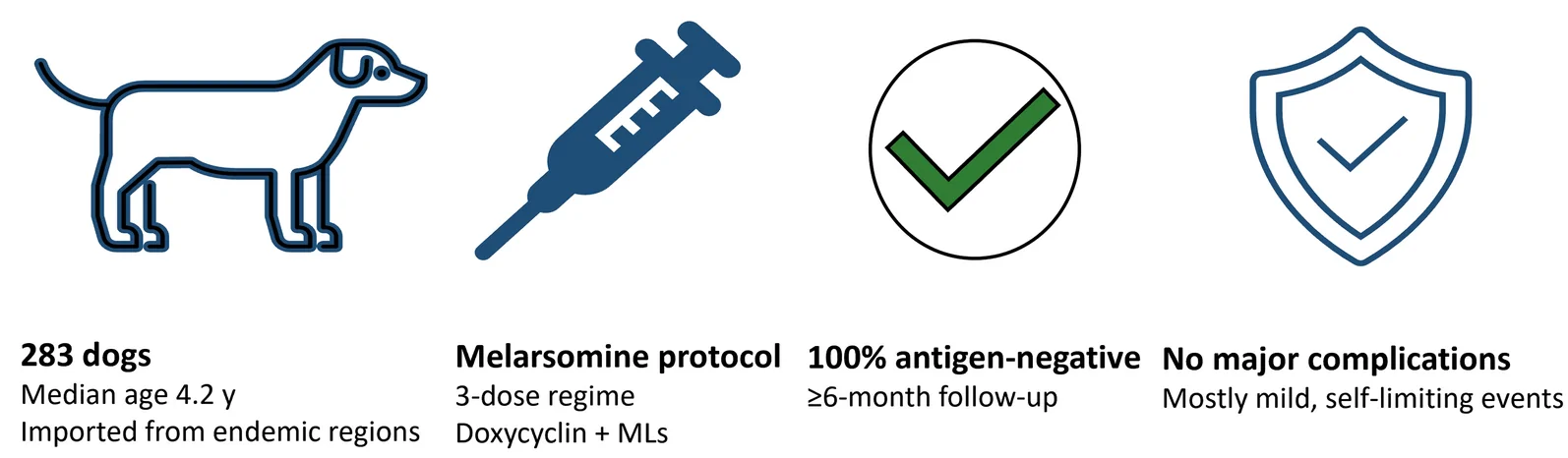

**Supplementary Information:**

The online version contains supplementary material available at 10.1186/s13071-026-07404-2.

## Background

In several Southern and Eastern European countries, including Romania and Hungary, large populations of free-roaming or stray dogs contribute to sustained transmission of *Dirofilaria* spp., as documented in recent epidemiological surveys [1–7]. Increasing numbers of these dogs are rehomed to non-endemic countries such as Germany. This trend not only raises ethical and economic questions but also presents substantial veterinary challenges, as many imported dogs arrive with pre-existing health conditions or carry infectious agents uncommon in Germany. One such agent is *Dirofilaria immitis*, a vector-borne filarial nematode and the causative agent of canine heartworm disease [4, 6, 8–10].

Although Germany is currently classified as non-endemic, the rehoming of dogs from endemic regions, combined with climate change expanding the range of competent mosquito vectors, has led to a notable increase in diagnosed cases [4, 11, 12]. Clinical experience shows that many rehomed dogs test positive for *D. immitis*, often with mild or absent clinical signs. Nevertheless, echocardiography reveals the presence of a substantial worm burden as well as diagnosis of pulmonary hypertension, underscoring the need for thorough staging and evidence-based therapy, even in apparently asymptomatic dogs [13–15].

Guidelines by the American Heartworm Society (AHS) and the European Society of Dirofilariosis and Angiostrongylosis (ESDA) recommend adulticide therapy with melarsomine in combination with macrocyclic lactones and doxycycline as the standard of care [1, 16]. Multiple studies have demonstrated the efficacy and acceptable safety profile of this protocol [17–19]. However, in non-endemic regions such as Germany, limited practical exposure to heartworm disease and concerns about treatment-related complications—often amplified by social media and non-expert commentary—have contributed to reluctance regarding the use of adulticide therapy, with lower-efficacy alternative regimens sometimes being preferred [20–22].

To address this gap, we retrospectively analyzed dogs treated at a German referral center in accordance with the AHS/ESDA protocol. Our study provides robust European evidence that severe complications are rare, thereby supporting guideline-based adulticide therapy using melarsomine and informing clinical practice in non-endemic settings.

## Methods

This study was a retrospective analysis of HWD cases presented to a German referral center between March 2017 and October 2024. Diagnosis was based on detection of *D. immitis* antigen using SNAP® 4Dx Plus (IDEXX Laboratories, Inc., Westbrook, ME, USA) and FASTest® HW Antigen (MEGACOR Diagnostik GmbH, Hoerbranz, Austria). Detection of microfilariae was performed either by the referring veterinarians or at the referral center. When microscopic examination was available, a modified Knott’s test was used for concentration and visualization of microfilariae [23]. Species differentiation was performed by acid phosphatase staining upon request. In addition, multiplex real-time PCR was used for detection of *D. immitis* DNA and *Dirofilaria* spp. DNA [24]. Because many dogs were initially tested by referring veterinarians, the diagnostic method (modified Knott vs. PCR) varied depending on local laboratory availability and clinical workflow. In dogs with negative antigen results but positive PCR for *D. immitis* microfilariae or echocardiographic visualization of worms, heat treatment of serum was performed to disrupt immune complexes [25–27]. Only dogs that subsequently underwent adulticide therapy with melarsomine at the referral center were included; animals were excluded if treatment was declined or carried out elsewhere.

For each dog, signalment was recorded, including breed, body weight, sex (male, neutered male, female, spayed female), and age. Country of origin and duration of residence in Germany were noted, and information on prior administration of macrocyclic lactones (ML) was collected. Dogs commonly received milbemycin or ivermectin in their country of origin, whereas moxidectin was used after diagnosis because it is the only ML licensed for microfilaricidal use in Germany. Concurrent diseases were documented, including other vector-borne infections such as babesiosis (PCR positive), leishmaniosis (antibody detection), and *Dirofilaria repens* (microfilarial PCR if applicable). Clinical signs at presentation were categorized as respiratory (coughing, panting, tachypnea), exercise intolerance, lethargy, gastrointestinal (vomiting, diarrhea), or other.

All dogs underwent diagnostic staging prior to adulticide therapy. Thoracic radiographs were performed either at the clinic or by the referring veterinarian, with orthogonal views preferred. Echocardiography (Vivid E9 or Vivid E95, GE Healthcare, Chicago, IL, USA) was performed on the day of diagnosis or staging and was repeated when feasible, no earlier than 6 months after the third melarsomine injection. Examinations were conducted by cardiologists following a systematic protocol. A comprehensive echocardiographic assessment was performed, encompassing both left and right heart parameters, with particular focus on the right pulmonary artery distensibility index (RPADi) and visualization of parallel linear structures (“double lines”) consistent with adult worms. Pulmonary hypertension (PH) was classified according to RPADi values as normal (> 35%), mild (28–35%), moderate (23–27%), or severe (< 23%), with the average of three measurements obtained [13–15, 28–30].

Worm burden was assessed echocardiographically and categorized as absent (score 1), few worms in the distal right pulmonary artery (RPA) (score 2), worms occupying the RPA and extending into the main pulmonary artery (MPA) (score 3), worms occupying the RPA and MPA up to the level of the pulmonary valves (score 4a) [15], or worms present in the right heart without evidence of caval syndrome (score 4b).

The severity of HWD was staged according to Maxwell et al. [18]. Class I included dogs without clinical signs and without abnormal findings on physical examination or imaging. Class II comprised dogs with mild clinical signs such as cough or exercise intolerance, accompanied by mild pulmonary infiltration, pulmonary arterial dilation, and mild pulmonary hypertension. Class III encompassed dogs with persistent coughing and exercise intolerance, along with marked pulmonary infiltration, significant pulmonary arterial dilation, or moderate-to-severe pulmonary hypertension. No dog with caval syndrome was included in the study.

Adulticide treatment followed the recommendations of the AHS [16] and ESDA [1]. A three-dose melarsomine regimen was used, with each dose injected deep intramuscularly into the lumbar muscles at 2.5 mg/kg (Immiticide®, Boehringer Ingelheim, Ingelheim am Rhein, Germany). All dogs received 7.5–10 mg/kg doxycycline orally twice daily (Doxybactin®, Dechra Regulatory B.V., distributed in Germany by Dechra Veterinary Products Deutschland GmbH) for 28 days and macrocyclic lactones topically or orally (Advocate® Spot-on, Bayer Animal Health, Leverkusen, Germany, or Simparica Trio®, Zoetis, Parsippany, NJ, USA) once every 28 days after HWD had been confirmed [31, 32]. In dogs that had resided in Germany for > two months, the protocol was shortened, with the first melarsomine dose administered 1 week after completion of doxycycline [33]. The second injection was scheduled 1 month later, and the third 24 h after the second.

Institutional modifications to the guidelines included pre-injection sedation to improve patient comfort and handling. Because most dogs in this cohort had been recently rehomed and often presented with marked anxiety, stress, or limited trust in unfamiliar handlers, light sedation was used to reduce fear-related reactions and facilitate safe positioning for deep intramuscular injection. Achieving brief muscle relaxation also minimized involuntary movement during the procedure and is expected to reduce post-injection muscle soreness. Dogs received 10–30 mg/kg gabapentin orally (Gabapentin STADA®, STADA Arzneimittel AG, Bad Vilbel, Germany) 2 h prior to arrival at the clinic. An intravenous catheter was placed, and propofol (Narcofol® 10 mg/ml, CP-Pharma Handelsgesellschaft mbH, Burgdorf, Germany) was administered intravenously by slow titration to effect. This technique allowed the use of a markedly low total dose (approximately 0.2–0.4 ml/kg), sufficient to achieve brief muscle relaxation without deep sedation, with an expected duration of 3–5 min. Following intramuscular melarsomine injection, patients were observed for 1 to 3 h, received intravenous fluids (Ringer Lactate, 5–10 ml/kg/h; Hartmann’s Lactated Ringers Solution, B. Braun Vet Care GmbH, Melsungen, Germany), and maropitant (1 mg/kg once intravenously; Cerenia®, Zoetis Inc., Parsippany, NJ, USA, or Prevomax®, Dechra Veterinary Products GmbH, Aulendorf, Germany). Meloxicam (0.1–0.2 mg/kg once intravenously; Metacam®, Boehringer Ingelheim Vetmedica GmbH, Ingelheim, Germany) was administered unless dogs were already receiving prednisolone. Prednisolone (0.5 mg/kg twice daily for 3 to 5 days, tapered to once daily for 3 to 4 weeks (Prednisolone JENAPHARM®, Jena, Germany) was prescribed in dogs with clinical signs, moderate-to-high worm burden, or elevated C-reactive protein. Sildenafil (2 mg/kg orally three times daily; STADA Arzneimittel AG, Bad Vilbel, Germany, or 1A Pharma GmbH, Holzkirchen, Germany) was administered to dogs with moderate-to-severe pulmonary hypertension or right atrial macrofilariae [29, 34]. Clopidogrel (loading dose 10 mg/kg orally, followed by 2 mg/kg once daily; Clopidogrel Zentiva®, S.C. Zentiva S.A., Bucharest, Romania) was administered to dogs with suspected pulmonary thromboembolism and discontinued 5 to 7 days before intramuscular melarsomine injections. Enoxaparin (1 mg/kg subcutaneously every 8 h; Clexane® multidose, Sanofi-Aventis Deutschland GmbH, Frankfurt am Main, Germany) was used in the same context and stopped 1 day prior to melarsomine administration to minimize the risk of hemorrhage and inadvertent intravenous injection [35, 36].

Owners were advised to maintain strict exercise restrictions beginning at the time of diagnosis and continuing until 3–4 weeks after the third melarsomine injection (Additional file 1).

At each point of owner contact—including the visit prior to the second melarsomine injection, the appointment for post-treatment microfilarial testing (or telephone discussion of results when testing was performed by the referring veterinarian), and the long-term follow-up visit ≥ 6 months after the third injection—owners were asked a set of questions regarding pain at the injection site, respiratory signs, gastrointestinal symptoms, lethargy, and any other abnormalities (Additional file 2). If clinical signs persisted, owners were instructed to contact the clinic by email.

As Germany is a non-endemic region where year-round heartworm prophylaxis is not routinely used, macrocyclic lactones were discontinued 4 to 8 weeks after the third melarsomine injection. To avoid false-negative microfilaria results, owners were instructed not to administer macrocyclic lactones for 30 days prior to follow-up testing. Microfilarial testing was performed no earlier than 30 days after discontinuation of prophylaxis.

Follow-up examinations were performed no earlier than 6 months after completion of adulticide therapy to assess treatment success and pulmonary hypertension. Antigen testing and microfilarial PCR were performed, with repeat heat treatment applied to dogs that had previously undergone heat treatment. Treatment success required both negative antigen and microfilaria tests no earlier than 6 months after the third melarsomine injection and the absence of macrofilariae on follow-up echocardiography. Echocardiography was performed in every dog at reexamination, regardless of initial pulmonary hypertension status, with careful evaluation of the MPA and RPA to rule out residual double linear echoes indicative of adult worms.

Dogs co-infected with *D. repens* received monthly moxidectin for 12 months, and microfilarial testing was repeated 30 days after treatment completion; all dogs with available follow-up tested negative.

## Statistics

Descriptive statistics were used to summarize demographic, clinical, diagnostic, and outcome variables. Continuous variables were summarized as median and range, and categorical variables as counts and percentages. Because the study aimed to characterize the cohort and treatment outcomes without testing predefined hypotheses, no inferential statistical analyses were conducted. Data were compiled, summarized, and subjected to basic descriptive statistical analysis using Microsoft Excel (Microsoft Corp., Redmond, WA, USA).

## Results

A total of 283 dogs with heartworm disease met the inclusion criteria for this retrospective study. The median age was 4.2 years (range: 8 months–11.5 years), and the median body weight was 19 (range: 3.4–51.1) kg. Females accounted for 53% (*n* = 156), of which 84% were spayed, while males comprised 45% (*n* = 127), of which 89% were neutered. All dogs originated from endemic European countries (Table 1). The duration of residence in Germany prior to clinical assessment was 6.2 months (range, 1 day to 72 months).

**Table 1:** Country of origin of dogs diagnosed with *Dirofilaria immitis* infection in Germany

Country of origin	Number of dogs	%
Romania	105	37.1
Hungary	62	21.9
Greece	25	8.8
Spain	21	7.4
Bulgaria	11	3.9
Portugal	8	2.8
Slovakia	7	2.5
Croatia	5	1.8
Ukraine	4	1.4
Sardinia	4	1.4
Macedonia	2	0.7
Russia	2	0.7
Turkey	2	0.7
Others	25	8.8

A positive antigen test served as the primary inclusion criterion. Eight dogs (2.8%) initially tested negative for antigen. In three of these, microfilarial PCR was positive, and in five, echocardiography revealed double linear structures consistent with adult worms. Following heat treatment of the sample, all eight dogs converted to antigen-positive, thereby confirming infection, despite the initial negative antigen tests.

Results of microfilarial testing were available for 256 dogs (90%), of which 153 (54%) were positive. Microfilarial PCR was performed in 129 dogs, with 96 (74%) testing positive for *D. immitis*, 25 (19%) positive for both *D. immitis* and *D. repens*, and 8 (6%) positive for *D. repens* alone. Macrocyclic lactones had been administered to 73% of dogs prior to presentation at the clinic.

The most common comorbidity was degenerative mitral valve disease, observed in 16 dogs (5.7%). Neoplastic conditions were observed in 10 dogs (3.5%), including cutaneous mast cell, mammary, thyroid, and transmissible venereal tumors. Neurological disorders were present in several cases: distemper-associated tremors in three dogs (1.1%), dental changes suggestive of prior distemper infection in one dog (0.4%), and other neurological signs such as back pain in two dogs (0.7%) and seizures in two dogs (0.7%). Additional individual findings included a third-degree atrioventricular block in one dog (0.4%), impaired wound healing, pyometra, and head trauma.

Other vector-borne infections were identified within the cohort. Babesiosis was confirmed by PCR in 8 dogs (2.8%), while leishmaniosis was diagnosed in 17 dogs (6.0%).

At the time of presentation, 150 dogs (53%) were asymptomatic; most of them had been recently adopted. Among symptomatic cases, the predominant findings were respiratory signs in 88 dogs (31%), followed by exercise intolerance in 42 dogs (15%) and gastrointestinal signs in 24 dogs (8.5%). Other clinical signs, including miscellaneous presentations, were observed in six dogs (2%).

Echocardiographic evaluation of worm burden identified macrofilariae in 224 dogs (79%). Table 2 summarizes the distribution of worm burden categories according to the echocardiographic scoring system. In three dogs (1.1%), macrofilariae were visualized within the right atrium, but without evidence of caval syndrome. These dogs were treated with sildenafil, which led to dislocation of the worms into the pulmonary arteries prior to initiation of melarsomine therapy [29, 34]. No complications occurred during adulticide treatment in these cases.

**Table 2:** Echocardiographic scoring system for worm burden and distribution in the right pulmonary artery (RPA) and main pulmonary artery (MPA), with number and percentage of affected dogs

Class	Description	Number of affected dogs	%
1	No worm visualized	60	21
2	Few worm echoes in the distal part of RPA	112	40
3	Worm echoes occupying RPA and extending to MPA	55	19
4a	Worm echoes occupying the whole RPA and MPA to the level of pulmonary valves	53	19
4b	Worm echoes identified in the right atrium (not causing cava syndrome)	3	1

RPADi for assessment of pulmonary pressures was available in 277 dogs. Of these, 130 (47%) had normal values, 97 (35%) mild, 38 (14%) moderate, and 12 (4%) severe pulmonary hypertension.

Based on clinical and imaging staging, 113 dogs (40%) were classified as Class I, 147 dogs (52%) as Class II, and 20 dogs (7%) as Class III.

Adverse events following melarsomine injections were generally mild, though occasional moderate or severe complications occurred. An overview of adverse reactions compared with previously published studies is provided in Table 3. After the first injection, 40 dogs (14%) developed self-limiting coughing or panting between days 5 and 10, presumed to be associated with worm death. Lethargy within the first days was reported in 31 dogs (11%), and injection-site soreness was documented in 27 dogs (9.5%). Gastrointestinal signs were observed in 11 dogs (3.9%) despite supportive therapy with intravenous fluids and maropitant.

**Table 3:** Adverse reactions following melarsomine treatment in this study compared with the Immiticide® product information [37] and previously published studies [17–19]. Values represent percentages unless otherwise indicated. GI, gastrointestinal; D, diarrhea; Resp., respiratory; V, vomiting; 1st, first injection; 3rd, third injection

Reaction	This study (1st/3rd)	Immiticide®	Maxwell et al. [18]	Romito et al. [19]	Still et al. [17] (1st/3rd)
Dogs included	283	311	50	35	539
Injection side reaction	9.2/2.1	32.8	10	2.9	31/35
GI signs	3.5/0.7	5.1(V) 2.6 (D)	24	2.9	20/21
Resp. signs	12.7/5.3	22.2	48	5.7	16/14
Behavioral	–	15.4	18	11.4	27/23
Mortality rate	0.7		14	0	1.3

Moderate complications occurred in four dogs (1.4%) following the first injection. One dog required in-hospital supportive care due to vomiting and diarrhea 2 days after treatment. Another presented after 18 days with lethargy, vomiting, and increased liver enzyme activity, necessitating hospitalization. A third dog with vomiting and elevated liver enzymes was managed as an outpatient. A fourth dog developed panting and lethargy 14 days post-injection, which resolved spontaneously. All dogs recovered, but the second injection was postponed by 7–10 days compared with the original schedule.

Following the second and third injections, gastrointestinal signs predominated, occurring in 21 dogs (7.4%). Coughing was reported in 15 dogs (5.3%), while injection-site pain or soreness was noted in 6 (2.1%). One dog presented 4 days after the third injection with acute dyspnea and cyanosis. Pulmonary thromboembolism was suspected but not confirmed; the dog recovered with in-hospital supportive care including the combination of clopidogrel and enoxaparin.

Two dogs (0.7%) died during the treatment course, though attribution to melarsomine remained uncertain. One dog developed pancytopenia 5 days after the third injection and was euthanized at the owners’ request despite potential responsiveness to transfusion therapy. The other experienced cardiac arrest under general anesthesia for dental extraction performed at the time of the second injection. These fatalities occurred during the treatment period but were not conclusively attributable to melarsomine. Overall, adverse events were predominantly mild and manageable, with only isolated moderate or severe complications, underscoring the safety of melarsomine administration in this cohort.

Follow-up examinations were performed in 221 dogs (78%). No heartworm antigen was detectable when dogs were retested at least 6 months after the last melarsomine injection, indicating 100% efficacy in this group. Microfilariae were likewise absent at reexamination.

Six dogs (2.1%) died prior to follow-up. Two of these cases have been described above; the remaining four dogs (1.4%) were put to sleep because of to malignant neoplasia, advanced neurological disease, or severe aggression unrelated to HWD. A total of 56 dogs (19.8%) were lost to follow-up However, telephone follow-up was possible for ten dogs (3.5%), whose owners reported that the animals were clinically well.

## Discussion

This study represents the largest European cohort of dogs with heartworm disease treated according to the AHS/ESDA protocol in a non-endemic country [1, 16]. By analyzing 283 rehomed dogs managed at a German referral center, we provide outcome data that complement existing reports from North America and Southern Europe. The institutional adaptations applied in this cohort, including pre-injection sedation, supportive therapy, and systematic echocardiographic staging, illustrate that guideline-based adulticide therapy is feasible in referral practice in a non-endemic region.

This retrospective study demonstrates that adulticide therapy with melarsomine, administered according to AHS/ESDA guidelines, is highly effective and generally well tolerated in dogs rehomed from endemic regions to Germany.

Microfilarial detection in this cohort relied on a combination of modified Knott’s testing and PCR, reflecting the diagnostic variability inherent in a referral population. Many dogs were initially tested by referring veterinarians, and PCR was frequently used because it is widely available in commercial laboratories in Germany, requires no specialized microscopy skills, and provides species differentiation without additional staining [26, 38, 39]. In contrast, modified Knott’s testing is not routinely performed in all primary care practices. Confirmation of microfilarial clearance is recommended even when preventives are administered, as these products do not consistently eliminate circulating microfilariae. Consequently, some dogs may require additional microfilaricidal treatment. In this cohort, a variety of MLs were used, and not all have consistent microfilaricidal effects [20, 40]. Therefore, we performed microfilarial PCR at follow-up to ensure that circulating microfilariae were absent regardless of the preventive previously administered [38].

Because Germany is a non-endemic region where dogs are not maintained on continuous prophylaxis, recent administration of macrocyclic lactones could transiently suppress circulating microfilariae; therefore, owners were instructed to withhold preventives for 30 days before follow-up testing to avoid false-negative results.

In dogs that had resided in Germany for > 2 months, we shortened the interval between doxycycline administration and the first melarsomine injection. Unlike in the US, where the AHS protocol assumes continuous exposure to infective mosquitoes and year-round macrocyclic lactone prophylaxis [16], dogs in Germany are not yet at risk of reinfection after several weeks in the country. Experimental studies show that L3 larvae molt to L4 within days and reach the pulmonary arteries as immature adults approximately 70–90 days after infection; therefore, all tissue stages present at the time of rehoming would have completed migration to the vasculature within 2 months. This eliminates the concern that very young larvae might still be migrating or below the age of susceptibility to melarsomine [20, 40–42]. Although approximately 90% of *Wolbachia* reduction occurs during the first 30 days of doxycycline therapy, additional reduction continues during days 30–60, as demonstrated by Chu et al. (2025). This progressive decline provides the rationale for maintaining an interval between doxycycline administration and melarsomine [32, 43–45]. In clinically stable dogs already living in a non-endemic region, this recovery period may be achieved during the 4-week doxycycline course itself. Our approach is further supported by Carretón et al., who demonstrated in an endemic setting that a shortened pre-treatment interval did not compromise efficacy or increase complications but improved owner compliance [33]. ESDA guidelines do not explicitly mandate a 2-month waiting period, and in the context of a non-endemic country, a shorter interval allows earlier adulticide administration without increasing clinical risk [1].

The manufacturer’s original recommendation for Immiticide® (melarsomine dihydrochloride) distinguished between dogs with limited clinicopathological abnormalities (Class I/II), treated with two injections 24 h apart, and those with marked abnormalities (Class III), treated with a three-dose regimen (one injection followed a month later by two injections 24 h apart) [37]. This staged approach was intended to reduce thromboembolic complications [32, 43, 46]. Subsequent consensus guidelines from the AHS and ESDA recommended a uniform three-dose protocol for all dogs, combined with doxycycline and monthly macrocyclic lactones. This change reflects both improved safety and higher efficacy, and our study confirms that the three-dose regimen is feasible and effective in a European referral setting.

Most dogs in this cohort were asymptomatic (53%), underscoring the importance of routine screening, particularly in endemic regions and in rehomed dogs. Among symptomatic cases, respiratory signs were most prevalent (31%), consistent with the pulmonary pathology associated with adult heartworm infection. Exercise intolerance (15%) and gastrointestinal signs (8.5%) were less common. In our cohort, RPADi identified pulmonary hypertension in more than half of the dogs, including 18% with moderate-to-severe changes. To our knowledge, this is the first large European study to systematically quantify pulmonary hypertension using RPADi in dogs with heartworm disease undergoing adulticide treatment with melarsomine. Diagnostic imaging is not always available prior to treating dogs with HWD, especially in endemic regions. Our findings therefore add complementary cardiological details and highlight that significant pulmonary vascular involvement can be present even in dogs with minimal or absent clinical signs, underscoring the importance of thorough echocardiographic staging [13–15].

Previous outcome studies of melarsomine therapy by Maxwell et al. (2014), Romito et al. (2023), and Still et al. (2024) concentrated on clinical signs, adverse events, and survival [17–19]. In this context, we evaluated treatment outcomes, complications, and adjunctive measures in our cohort.

Post-treatment complications in our study were generally mild and self-limiting. Injection-site soreness and transient coughing were the most frequently observed adverse effects, particularly after the first dose. Severe complications were uncommon (0.7%) but clinically relevant, including one case of cardiac arrest during concurrent dental anesthesia and one case of pancytopenia following the third injection. These events underscore the importance of careful patient selection and monitoring, especially when elective procedures are scheduled close to adulticide administration. Overall, melarsomine remained safe and effective when used according to recommended protocols, and clinicians should communicate potential adverse effects clearly while emphasizing the need for post-treatment rest and follow-up care.

Additional treatment modifications in our cohort included sedation with gabapentin and propofol, intravenous fluids, maropitant, meloxicam or prednisolone, sildenafil for pulmonary hypertension or intracardiac macrofilariae [29, 34], and clopidogrel and/or enoxaparin for suspected thromboembolism [35, 36]. These treatments were intended to minimize soreness, reduce complications, and stabilize patients with advanced disease. Comparable strategies have been reported internationally: Maxwell et al. (2014) used sedation to facilitate injection and noted behavioral changes likely linked to pain [18]; Romito et al. (2023) reported sedation in 23% of dogs, ice application to injection sites in 86%, and hospitalization for 6–48 h to ensure cage rest [19], while Still et al. (2024) employed trazodone, butorphanol/dexmedetomidine, and tramadol for analgesia, accompanied by 2.5–4.5 h of in-clinic monitoring after each injection [17]. Taken together, these reports suggest that adjunctive care—including sedation, analgesia, and short periods of post-injection monitoring—may offer meaningful benefits and merits consideration as part of heartworm therapy.

In interpreting our clinical outcomes, it is important to consider that all dogs in this cohort were managed under exercise-restriction instructions (Additional file 1). Because increased physical activity can elevate pulmonary blood flow and dislodge degenerating worms, consistent restriction of vigorous activity is a key factor in reducing the risk of pulmonary thromboembolism during adulticide therapy. Clear guidance and early implementation of these measures may have contributed to the generally favorable tolerance of treatment observed in our population, particularly given that many dogs were recently rehomed and presented with variable baseline stress levels and limited prior medical oversight [13].

Our findings are consistent with recent international data (Table 3). Romito et al. (2023) reported rare, mild, and rapidly resolving adverse effects in 35 dogs treated in Italy, all of which survived therapy [19]. Still et al. (2024) described similarly favorable outcomes in 556 dogs treated in a high-volume outpatient setting, with 97% completing therapy, 99% achieving antigen negativity at 9 months, and a mortality rate of 1.3% [17]. In contrast, Maxwell et al. (2014) documented a higher mortality rate (14%) in a cohort that included dogs with advanced disease (Class III/IV) and caval syndrome [18]. Differences in case selection likely explain this variation, as Romito, Still, and our study focused on Class I–III dogs without caval syndrome. Across all studies, adverse events were common but predominantly mild, and treatment efficacy was consistently high.

This retrospective study has several limitations. It was conducted at a single referral center, which may restrict the generalizability of the findings to primary care settings or other European regions. Follow-up data were incomplete, as approximately one fifth of dogs were lost to follow-up, and outcome information for these cases relied partly on owner reports rather than standardized clinical re-examination. Prior administration of macrocyclic lactones in many dogs may have influenced diagnostic sensitivity, particularly for microfilarial detection, and could have resulted in false-negative results. Echocardiographic assessment of worm burden and pulmonary hypertension was analyzed by a single cardiologist, ensuring consistency but precluding evaluation of inter-observer variability. Finally, adverse events were documented from medical records and owner reports, which may underestimate mild or transient reactions.

Despite these limitations, this study has notable strengths. It provides a uniquely comprehensive dataset on guideline-based adulticide therapy in a non-endemic European referral setting. The systematic collection of demographic, diagnostic, and treatment data, combined with consistent echocardiographic staging, provides a robust dataset. These institutional modifications further enhance clinical relevance of the findings and offer practical insights for referral centers and general practitioners managing rehomed dogs.

Our data provide several clinically important insights for veterinarians in nonendemic regions who need to deal with heartworm disease. Notably, more than half of the dogs in this cohort were asymptomatic at presentation, despite many having echocardiographic evidence of worm burden and pulmonary hypertension. This underscores that apparent clinical normality is a poor surrogate for cardiopulmonary involvement and that clinical signs alone may substantially underestimate disease severity. Thorough staging therefore is recommended, even in apparently healthy animals.

The excellent treatment response observed in this cohort reflects the effectiveness of guideline-based adulticide therapy. Supportive measures such as sedation, intravenous fluids, and standardized monitoring were incorporated to reduce discomfort and mitigate complications and may explain the low frequency of severe adverse events observed.

## Conclusion

Adulticide treatment with melarsomine, administered according to current AHS/ESDA guidelines, proved highly effective and well tolerated in this large European cohort of dogs with heartworm disease. Most adverse events were mild and self-limiting, and severe complications were rare. Importantly, all dogs re-examined ≥ 6 months after therapy tested antigen-negative, underscoring the reliability of the three-dose protocol in a non-endemic setting. These findings provide robust evidence that guideline-based adulticide therapy can be safely implemented in European referral practice and support its use as the standard of care for dogs imported from endemic regions. Continued surveillance and further multicenter studies will help refine best practices and strengthen clinical decision-making for heartworm management across diverse European populations.

## Supplementary Information

Additional file 1: Client instruction for exercise restriction.

Additional file 2: Follow‑up questionnaire for monitoring clinical signs

## Data Availability

Data supporting the main conclusions of this study are included in the manuscript.

## Abbreviations

AHS: American Heartworm Society
ESDA: European Society of Dirofilariosis and Angiostrongylosis
HWD: Heartworm disease
MPA: Main pulmonary artery
RPA: Right pulmonary artery
RPADi: Right pulmonary artery distensibility index

## Declarations

### Use of generative AI and AI-assisted technologies in the writing process

The authors used an AI-based language tool (Microsoft Copilot) to assist with English phrasing, grammar, and clarity during manuscript preparation. The authors reviewed, edited, and approved all content, and take full responsibility for the integrity and accuracy of the manuscript.
