# Supplementary material for: Adulticide treatment with melarsomine: outcome in 283 heartworm-positive dogs in Germany
Source: Parasit Vectors. 2026 Aug 20;19:356. doi: 10.1186/s13071-026-07404-2 (PMC13491706; doi:10.1186/s13071-026-07404-2)
Supplement: Supplementary file 1 [file 13071_2026_7404_MOESM1_ESM.docx]

**Supplementary Material 1: Client Instructions for Exercise Restriction**

Owners received standardized written and verbal instructions regarding strict exercise restriction following confirmation of heartworm disease. The rationale provided was that increased heart rate and pulmonary blood flow may dislodge degenerating or migrating worms, potentially leading to pulmonary thromboembolism.

**General principles**

- Exercise restriction began immediately upon a positive antigen or microfilaria test.
- Dogs were to avoid all vigorous activity, including running, playing, jumping, and situations that provoke excitement or arousal.
- Outdoor activity was limited to brief, calm leash walks for elimination only.
- Owners were advised to prevent fence‑running, chasing stimuli, or high‑excitement greetings when visitors arrived.
- Attendance at dog parks, dog day‑care, group walks, or training classes was not permitted during the restriction period.

**Adaptation to clinical severity**

- **Mild infection:** Controlled leash walks and calm indoor activity were allowed; all vigorous activity was prohibited.
- **Moderate to severe infection or evidence of pulmonary hypertension:** Dogs were to be kept as quiet as possible within the home environment. Confinement to a single room was recommended for highly excitable dogs, but crate or kennel confinement was *not* advised due to stress concerns in recently rehomed animals.

**Duration of restriction**

- Exercise restriction continued throughout the adulticide treatment period and until approximately three to four weeks after the final melarsomine injection.

**Welfare and behavioural support**

Owners were encouraged to use low‑arousal mental stimulation to maintain welfare without increasing physical exertion. Examples included basic obedience training, clicker training, scent‑based games, and calm interactive activities. Weight monitoring was recommended due to reduced physical activity.
