# Supplementary material for: Adulticide treatment with melarsomine: outcome in 283 heartworm-positive dogs in Germany
Source: Parasit Vectors. 2026 Aug 20;19:356. doi: 10.1186/s13071-026-07404-2 (PMC13491706; doi:10.1186/s13071-026-07404-2)
Supplement: Supplementary file 2 [file 13071_2026_7404_MOESM2_ESM.docx]

**Supplementary Material 2: Follow‑up Questions**

Owners were asked a consistent set of questions at each point of contact during the treatment process. These contacts included: (1) the visit prior to the second melarsomine injection, (2) the appointment for post‑treatment microfilaria testing (or telephone discussion of results when testing was performed by the referring veterinarian), and (3) the long‑term follow‑up visit, which occurred no earlier than six months after the third melarsomine injection.

Owners were additionally instructed to contact the clinic by email if any clinical signs persisted for several hours; these reports were included in the dataset. These questions were used to document adverse events and clinical signs consistently across the follow‑up time points.

**1. Pain or discomfort**

- Did you observe any pain or swelling at the injection site?
- Did the dog show difficulty rising, climbing stairs, or moving normally?
- Were there any other signs of discomfort?
- If pain or discomfort was present, how long did it persist?
- Did pain management require veterinary treatment?

**2. Respiratory signs**

- Has the dog shown any coughing or increased respiratory rate?
- If coughing occurred:
  - On which day(s) after the injection did it begin?
  - Was the cough dry or moist?
  - How long did the cough persist?
- Did you observe any episodes of tachypnoea or laboured breathing?

**3. Gastrointestinal signs**

- Did the dog experience vomiting or diarrhoea?
- If yes, were these signs self‑limiting or did they require veterinary treatment?

**4. Lethargy**

- Did the dog appear unusually tired or otherwise lethargic?

**5. Other observations**

- Were there any additional abnormalities or concerns not covered above?
